# Targeting Super‐Enhancers via Nanoparticle‐Facilitated BRD4 and CDK7 Inhibitors Synergistically Suppresses Pancreatic Ductal Adenocarcinoma

**DOI:** 10.1002/advs.201902926

**Published:** 2020-02-16

**Authors:** Chen‐Song Huang, Xinru You, Chunlei Dai, Qiong‐Cong Xu, Fuxi Li, Li Wang, Xi‐Tai Huang, Jie‐Qin Wang, Shi‐Jin Li, Zhuoxing Gao, Jun Wu, Xiao‐Yu Yin, Wei Zhao

**Affiliations:** ^1^ Department of Pancreato‐Biliary Surgery The First Affiliated Hospital of Sun Yat‐sen University Guangzhou 510080 China; ^2^ Key Laboratory of Sensing Technology and Biomedical Instrument of Guangdong Province School of Biomedical Engineering Sun Yat‐sen University Guangzhou 510006 China; ^3^ RNA Biomedical Institute Sun Yat‐sen Memorial Hospital Sun Yat‐sen University Guangzhou 510120 China; ^4^ Key Laboratory of Stem Cells and Tissue Engineering (Sun Yat‐sen University) Ministry of Education Guangzhou 510080 China

**Keywords:** BRD4, CDK7, nanoparticles, pancreatic ductal adenocarcinoma, super enhancers

## Abstract

Pancreatic ductal adenocarcinoma (PDAC) is a lethal malignant cancer with complex genomic variations, and no targetable genomic lesions have been found yet. Super‐enhancers (SEs) have been found to contribute to the continuous and robust oncogenic transcription. Here, histone H3 lysine 27 acetylation (H3K27ac) is profiled in PDAC cell lines to establish SE landscapes. Concurrently, it is also shown that PDAC is vulnerable to the perturbation of the SE complex using bromodomain‐containing protein 4 (BRD4) inhibitor, JQ1, synergized with cyclin‐dependent kinase 7 (CDK7) inhibitor, THZ1. Formulations of hydrophobic l‐phenylalanine‐poly (ester amide) nanoparticles (NPs) with high drug loading of JQ1 and THZ1 (J/T@8P4s) are further designed and developed. J/T@8P4s is assessed for size, encapsulation efficiency, morphology, drug release profiles, and drug uptake in vitro. Compared to conventional free drug formulation, the nanodelivery system dramatically reduces the hepatotoxicity while significantly enhancing the tumor inhibition effects and the bioavailability of incorporated JQ1 and THZ1 at equal doses in a Gemcitabine‐resistant PDAC patient‐derived xenograft (PDX) model. Overall, the present study demonstrates that the J/T@8P4s can be a promising therapeutic treatment against the PDAC via suppression of SE‐associated oncogenic transcription, and provides a strategy utilizing NPs to assist the drug delivery targeting SEs.

## Graphical abstract

The H3K27ac profile defines super‐enhancer (SE) landscapes in pancreatic ductal adenocarcinoma (PDAC). PDAC is vulnerable to the perturbation of the SE complex using JQ1 synergized with THZ1. Importantly, the nanodelivery system significantly enhances the tumor inhibition effects of combination therapy on a Gemcitabine‐resistant PDAC patient‐derived xenograft model.

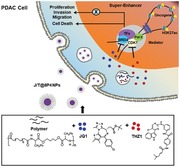

## 1 Introduction

Pancreatic ductal adenocarcinoma (PDAC), a painful and fatal malignancy with high mortality, accounts for more than 90% pancreatic cancer. Its incidence is increasing in recent years, and it is estimated that PDAC will be ranked as the second cause of cancer death in the world by 2020.^[^[qv: 1]^]^ Although genomic sequencing has improved the understanding of PDAC genetic abnormalities, few targetable targets have been found.^[^[qv: 2]^]^ There are no clinically available molecular targeting drugs for PDAC patients. A better understanding of the complex mechanisms underlying the progress of PDAC, such as the effect of epigenomic changes on the oncogenic transcription, is urgently needed for the development of effective treatment.

The hallmarks of cancer cells phenotypes (unlimited proliferation, replicative immortality, apoptosis evasion, and metastasis) are caused by aberrant regulation of gene expression. In PDAC, early reports demonstrated that the oncogenic transcription factors (TFs), such as SMAD3, were highly expressed and correlated with the malignant characteristics. However, the mechanism that mediates the aberrant transcription programs in PDAC is not fully understood. Super‐enhancers (SEs) are large clusters of enhancers typically exhibiting an enrichment of H3K27ac which are occupied by an extreme density of TFs and cofactors. SEs can specify cell identity and conform to cell type‐specific functions in normal cells. In many types of cancer cells, SEs are enriched in oncogenic TFs, which are responsible for tumor pathogenesis. The features make these cancer cells sensitive to SE complex disruption.^[^[qv: 3]^]^

BRD4 and CDK7 are components of SE complex acting as positive regulators of SE‐mediated transcription. BRD4 plays a key role in regulating chromatin remodeling and transcriptional activation. CDK7, a subunit of TFIIH, promotes efficient transcriptional initiation and elongation by phosphorylating the carboxy‐terminal domain of RNA polymerase II (Pol II). Recently, various studies have demonstrated the promising anticancer effects of the BRD4 inhibitor JQ1^[^[qv: 3b,4]^]^ or CDK7 inhibitor, THZ1^[^[qv: 5]^]^ by preferentially targeting the SE complex to affect carcinogenic transcription and tumor characteristics. Although JQ1 is a molecule that can be permeable to cells because of its high hydrophobicity, its half‐life is too short (about 1 h) to be used in clinical treatment. THZ1 has limited use in cancer therapy due to the adverse effects such as poor bioavailability, low cell specificity, and dose‐dependent cytotoxicity.

Preclinical and clinical studies have shown that nanoparticle (NP) loaded drugs have reduced toxicity and improved efficacy. Nab‐paclitaxel combined with Gemcitabine has been used as the first‐line therapy in the treatment of PDAC.^[^[qv: 6]^]^ However, NPs have never been reported for the delivery of drugs targeting SEs. We hypothesize that the use of nanocarrier may improve the targeted administration of JQ1 or THZ1 through reducing the dose of drugs needed by patients, stabilizing the drugs in serum, maximizing their therapeutic effect, and reducing the side effects.

**Scheme 1:**
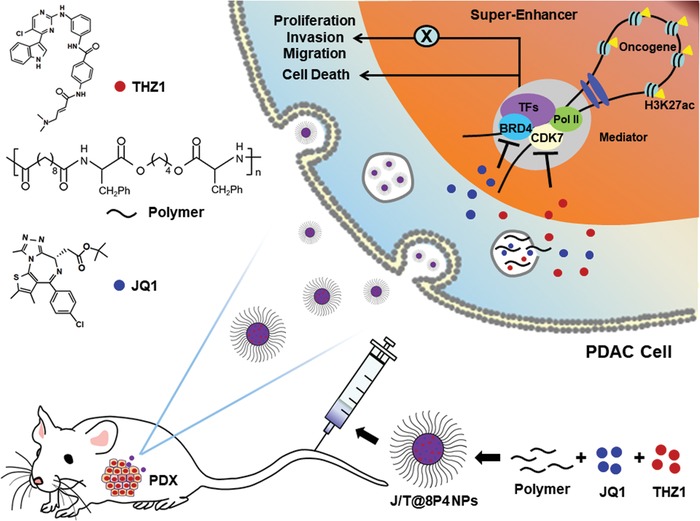
Schematic representation of preparation of J/T@8P4 NPs and how J/T@8P4 NPs suppress PDAC through targeting SE‐associated oncogenes.

Here, we developed and screened formulations of hydrophobic poly(ester amide) NPs conjugated with a synergistic combination of SE complex targeting agents as a potential basis for one such advanced platform for PDAC therapy (**Scheme** **1**). We report that JQ1, in combination with THZ1, is an effective molecular targeting therapeutic option for PDAC treatment. The use of a nanoenabled platform was essential for the controlled drug release and improved drug delivery. We prove the feasibility of achieving tumor eradication of the Gemcitabine‐resistant PDAC in the patient‐derived xenograft (PDX) model, using the nanodelivery approach of JQ1 and THZ1. In addition, the nanodrugs had the benefit of significantly reducing the liver damage, with boosting of intratumoral drug concentrations.

## 2 Results

### 2.1 Identification of SEs in PDAC Reveals Potential Therapeutic Targets

**Figure 1:**
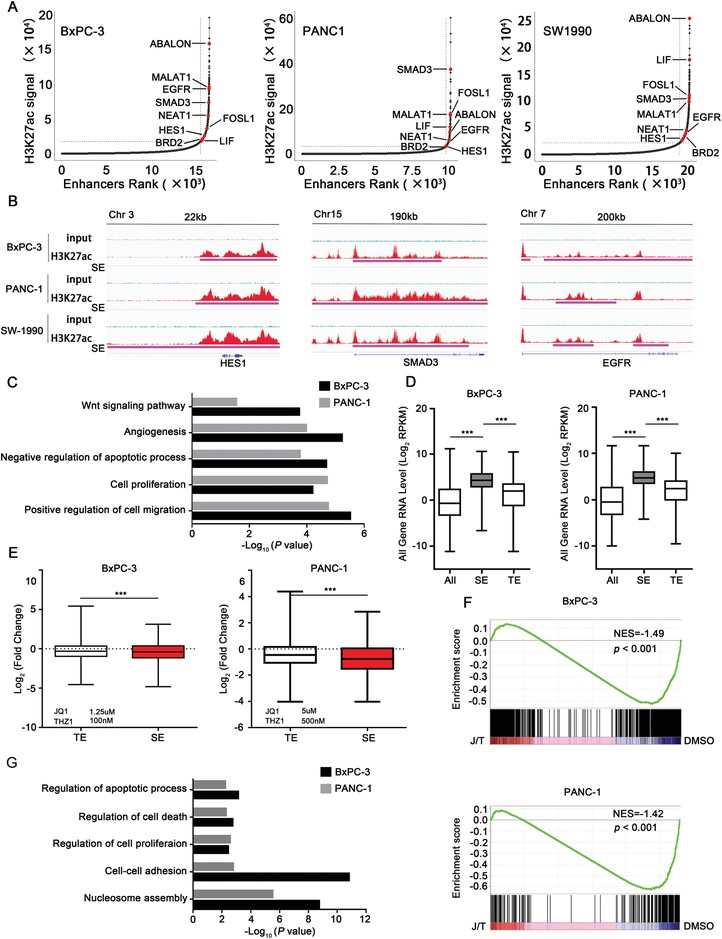
Characterizing the SE landscapes in PDAC cell lines. A) Enhancers ranked by H3K27ac ChIP‐seq signal over input. SE‐associated genes in all three PDAC cell lines are highlighted in red. B) H3K27ac ChIP‐seq profiles of representative SE‐associated gene loci (HES1, SMAD3, and EGFR) in BxPC‐3, PANC‐1, and SW‐1990 cells. C) GO analysis of SE‐associated genes in BxPC‐3 (870 SE genes) and PANC‐1 cells (305 SE genes). D) Box plots showing relative RNA expression levels of total enhancer (ALL), typical‐enhancer (TE), and SE‐regulated genes in BxPC‐3 and PANC‐1 PDAC cells. E) Box plots showing the fold changes of RNA expression levels of TE and SE‐regulated genes upon JQ1 and THZ1 co‐treatment (24 h). F) GSEA of the downregualted SE‐associated transcripts following JQ1 and THZ1 co‐treatment. G) GO analysis of the downregualted SE‐associated genes upon JQ1 and THZ1 co‐treatment in BxPC‐3 and PANC‐1 cells. Data are presented as mean ± SD. *p < 0.05, **p < 0.01, and ***p < 0.001 were calculated according to a Student's *t*‐test. All presented results are from three independent experiments.

The SE‐associated genes in cancer are enriched for drivers of carcinogenic state and key regulators of cell identity. We characterized SE landscape in three PDAC cell lines through H3K27ac ChIP‐seq. A total of 870, 305, and 1239 SE‐associated genes were identified in BxPC‐3, PANC‐1, and SW‐1990 cells, respectively (**Figure** **1**A). A number of the SE‐associated genes were markers of tumor cell proliferation, including *EGFR*. Many genes are involved in invasion and metastasis, such as *SMAD3*, as well as genes play critical roles during pancreatic development, such as *HES1* (Figure 1B). Gene ontology (GO) analysis was performed to further explore the functional implications of these SE‐associated genes in PDAC cells. Importantly, these SE‐associated genes were significantly enriched in GO terms of positive regulation of cell migration and angiogenesis, cell proliferation, and negative regulation of apoptotic process (Figure 1C).

When linked to gene expression quantified by RNA‐seq of BxPC‐3 and PANC‐1, we observed that RNA abundance of SE‐associated transcripts was significantly higher than that of typical enhancer (TE)‐associated transcripts in both PDAC cell lines (Figure 1D).

Combinatorial treatment of JQ1 and THZ1 resulted in the greatest downregulation of the expression of SE‐associated transcripts, but had less effect on TE‐associated transcripts (Figure 1E). In addition, gene set enrichment analysis (GSEA) also showed that SE‐associated transcripts were enriched for those genes that were sensitive to combined treatment of JQ1 and THZ1 (Figure 1F). We then performed GO analysis on all downregulated genes upon combined treatment and observed enrichment for cell–cell adhesion, regulation of cell proliferation, as well as regulation of cell death (Figure 1G). These results suggested that SE‐associated genes were sensitive to combined treatment of JQ1 and THZ1 treatment in PDAC.

### 2.2 Synergistic Effects of the BET Bromodomain Inhibitor JQ1 and CDK7 Inhibitor THZ1 against PDAC

**Figure 2:**
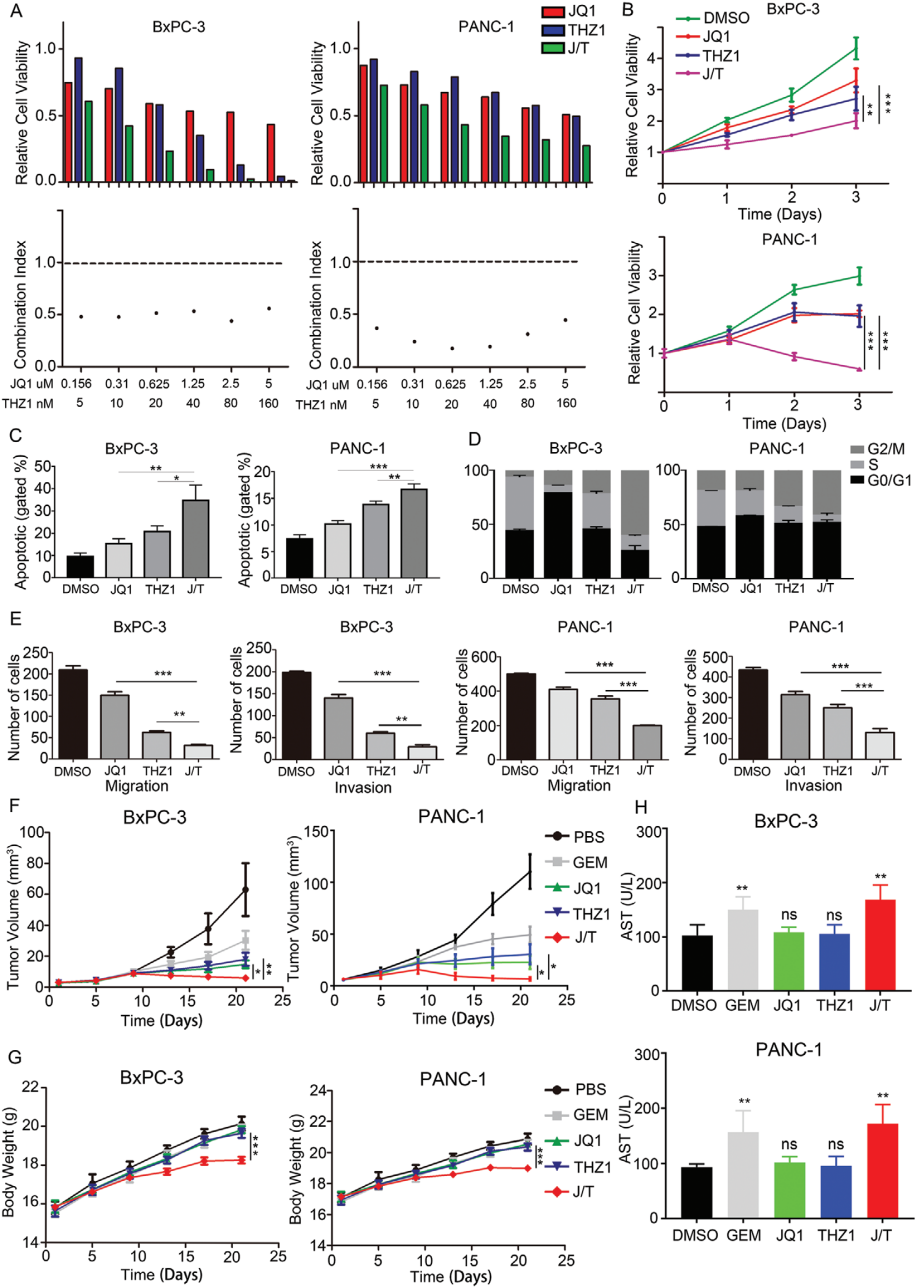
Synergistic effects of JQ1 and THZ1 against PDAC. A) Viability and synergy in JQ1 and THZ1 treatments. PDAC cultures treated with JQ1 and THZ1 individually or in combination at indicated concentrations for 48 h. Top) Cell viabilities were measured and normalized to DMSO control values (*n* = 3 wells per data point). Bottom) CI was calculated by using CalcuSyn software. CI less than 1 demonstrates synergy between two drugs. B) Cell viability assay showing the effects of JQ1 or/and THZ1 treatment on BxPC‐3 and PANC‐1 cells at indicated time points. C) Apoptosis analysis of BxPC‐3 and PANC‐1 cells treated with JQ1 or/and THZ1. D) Cell cycle analysis of BxPC‐3 and PANC‐1 cells treated with JQ1 or/and THZ1. E) Invasion and migration assays of BxPC‐3 and PANC‐1 cells treated with JQ1 or/and THZ1. F) Tumor growth curves of the mice (*n* = 6 per group) treated with PBS, Gemcitabine (50 mg kg^−1^, twice per week), JQ1 (50 mg kg^−1^, daily), THZ1 (10 mg kg^−1^, twice daily), and JQ1 (50 mg kg^−1^, daily) combined with THZ1 (10 mg kg^−1^, twice daily) for 21 days. The tumor volume was monitored every 4 day. G) Weight of tumors derived from mice (*n* = 6) in each group. H) Serum AST of mice (*n* = 6) in each group. Data are presented as mean ± SD. *p < 0.05, **p < 0.01, and ***p < 0.001 were calculated according to a Student's *t*‐test. All presented results are from three independent experiments.

We next sought to determine whether THZ1 had a synergistic effect with JQ1 on inhibition of PDAC viability. THZ1 and JQ1 combination robustly reduced PDAC cells (BxPC‐3 and PANC‐1) viability in a dose‐dependent manner, with low combination index (CI) at all concentrations, reflecting a high level of synergy in both PDAC cell lines (**Figure** **2**A and Figure S1, Supporting Information). Co‐treatment with THZ1 and JQ1 led to a more significant reduction in cell proliferation than JQ1 or THZ1 alone (Figure 2B), and was proved more effective in inducing apoptosis (Figure 2C and Figure S2, Supporting Information) and G2/M phase cell‐cycle arrest (Figure 2D). Synergistic inhibition of cell migration and invasion with the JQ1 and THZ1 combination therapy was also observed in BxPC‐3 and PANC‐1 cells (Figure 2E and Figure S3, Supporting Information).

Accordingly, mice bearing subcutaneous xenograft derived from BxPC‐3 or PANC‐1 cells showed significant reduction in tumor growth after treated with JQ1 and THZ1, meanwhile the JQ1 or THZ1 alone treatment showed the less effect (Figure 2F and Figure S4, Supporting Information). The effect of JQ1 and THZ1 combination therapy is also significantly better than that of Gemcitabine, the first‐line clinical drug in the treatment of PDAC. However, the weight of JQ1 and THZ1 co‐treated mice decreased significantly (Figure 2G), and the serum aspartate aminotransferase (AST) of these mice was markedly increased (Figure 2H), suggesting that JQ1 together with THZ1 are not sufficiently safe for clinical translation due to hepatotoxicity.

### 2.3 Formation of J/T@8P4 NPs with High Drug Loading and Controlled Release

**Figure 3:**
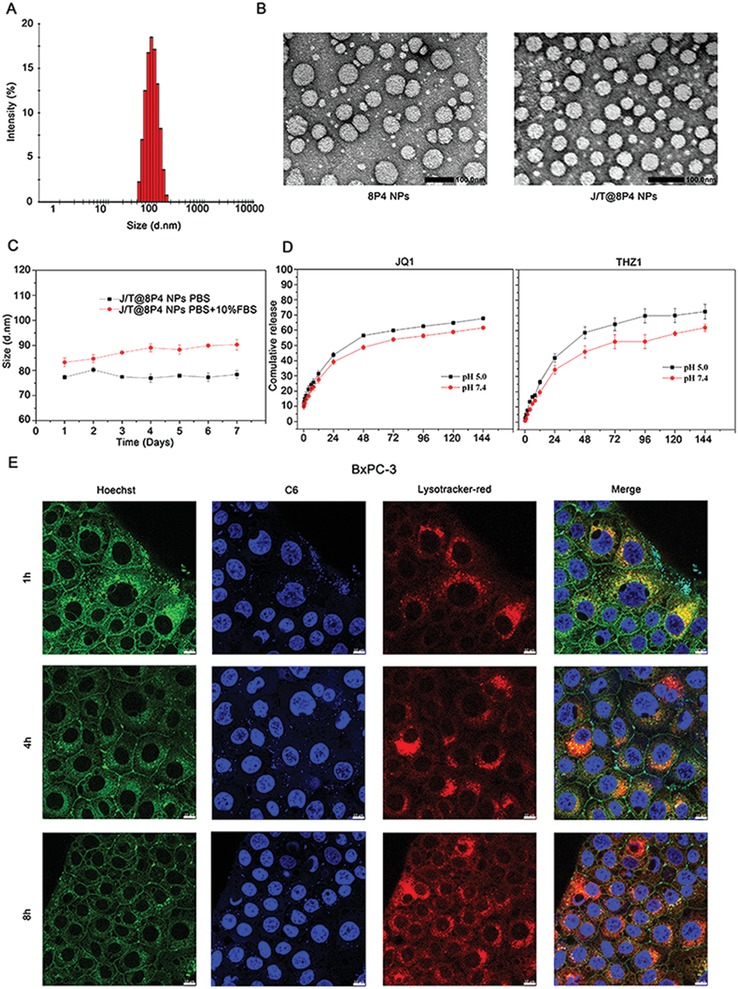
Characterization of J/T@8P4 NPs. A) Size distribution and B) representative TEM image (*n* = 3) of J/T@8P4 NPs. Scale bar, 100 nm. C) Stability of J/T@8P4 NPs in PBS and PBS + 10% FBS. D) Cumulative release profile of JQ1 or THZ1 from J/T@8P4 NPs. E) Representative CLSM images (*n* = 5) of BxPC‐3 cells treated with NP_C6_ for 1, 4, and 8 h. The lysosomes were labeled by Lyso‐Tracker. Scale bar = 10 µm. Data are presented as mean ± SD. *p < 0.05, **p < 0.01, and ***p < 0.001 were calculated according to a Student's *t*‐test. All presented results are from three independent experiments.

In order to improve the safety of combined treatment of JQ1 and THZ1, we here propose a radically different approach to evaluate the formulation of JQ1 and THZ1‐loaded nanodrug in a library of NPs with different physical and chemical properties. We created a library comprising hydrophobic l‐phenylalanine‐polymer NPs, poly(D,L‐lactic‐*co*‐glycolic acid)/poly(lactic acid) NPs, and l‐cystine‐polymer NPs (Figures S5 and S6, Supporting Information). We then study the stability and drug loading capability of different nanodrugs. Based on the results of this screening, we formulated 8P4 hydrophobic l‐phenylalanine‐poly(ester amide) NPs with the most effective drug loading capability (Tables S1 and S2, Supporting Information). The structure of 8p4 polymer was confirmed by ^1^H NMR and gel permeation chromatography (Figures S7 and S8, Supporting Information). Next, we explored whether the JQ1 and THZ1‐loaded 8P4 NPs (J/T@8P4 NPs) lead to more effective antitumor efficiency with better tolerance. The average size of J/T@8P4 NPs was 89.5 ± 2.53 nm with a narrow polydispersity index (**Table** **1**), indicating that the morphology of these nanodrugs was uniform and spherical (**Figure** **3**A,B). Therefore, the stability of J/T@8P4 NPs in phosphate‐buffered saline (PBS) and PBS containing 10% fetal bovine serum (FBS) was examined. The particle size of J/T@8P4 NPs was almost unchanged, fluctuating between 70 and 90 nm over 7 days (Figure 3C). Next, the in vitro release performance of J/T@8P4 NPs was investigated under pH 7.4 and pH 5.0.^[^[qv: 7]^]^ Ideally, the actual JQ1 or THZ1 release of J/T@8P4 NPs was promoted under acidic conditions (Figure 3D). In addition, J/T@8P4 NPs showed improved pharmacokinetic behavior compared with free drugs (Figure S9, Supporting Information).

**Table 1:** Characterization of J/T@8P4 NPs

Tested samples	Diameter [nm]^a)^	Polydispersity index^a)^	Zeta potential [mV]^a)^	LC of JQ1^b)^ [%]	EE of JQ1^b)^ [%]	LC of THZ1^b)^ [%]	EE of THZ1^b)^ [%]
J/T@8P4 NPs	89.5 ± 2.53	0.19 ± 0.03	32.23 ± 4.55	16.57 ± 0.25	66.29 ± 0.59	7.44 ± 0.63	89.15 ± 7.57

^a)^ Determined via DLS

^b)^ Analyzed via HPLC.

To explore the ability of controlled drug release of J/T@8P4 NPs in PDAC, we then examined the distribution of these nanodrugs in BxPC‐3 cells. Coumarin 6 (C6), instead of JQ1 or THZ1, was loaded into 8P4 to observe the intracellular distribution in BxPC‐3 cells. We used the Lyso‐Tracker Red probes to mark lysosomes. After incubation with fluorescent C6‐loaded NPs for 1 h, intracellular green fluorescence of C6 dramatically increased (Figure 3E), indicating that the drugs can be transported into the cells quickly. Meanwhile, we detected the intracellular accumulation of lysosomes and found green fluorescence of C6 could merge with red fluorescence of lysosomes, which implies NPs deliver drugs to lysosomes. Since the lysosomes are acidic organelles (pH 5.0–5.5), the 8P4 NPs may dissociate in lysosomes, thus triggering the release of loaded drugs. Indeed, we found the NPs markedly damaged the lysosomes after accumulation in lysosome for 8 h, resulted in the robust release of drug (C6) in the cells (Figure 3E), which may increase the drug concentrations in PDAC cells. These data confirm that the NPs are sensitive to the low pH and indicate that the drug can be concentrated in lysosomes and released instantly to effectively eliminate PDAC cells.

### 2.4 Cellular Uptake Behavior of the Dual Drug–Loaded 8P4 NPs

**Figure 4:**
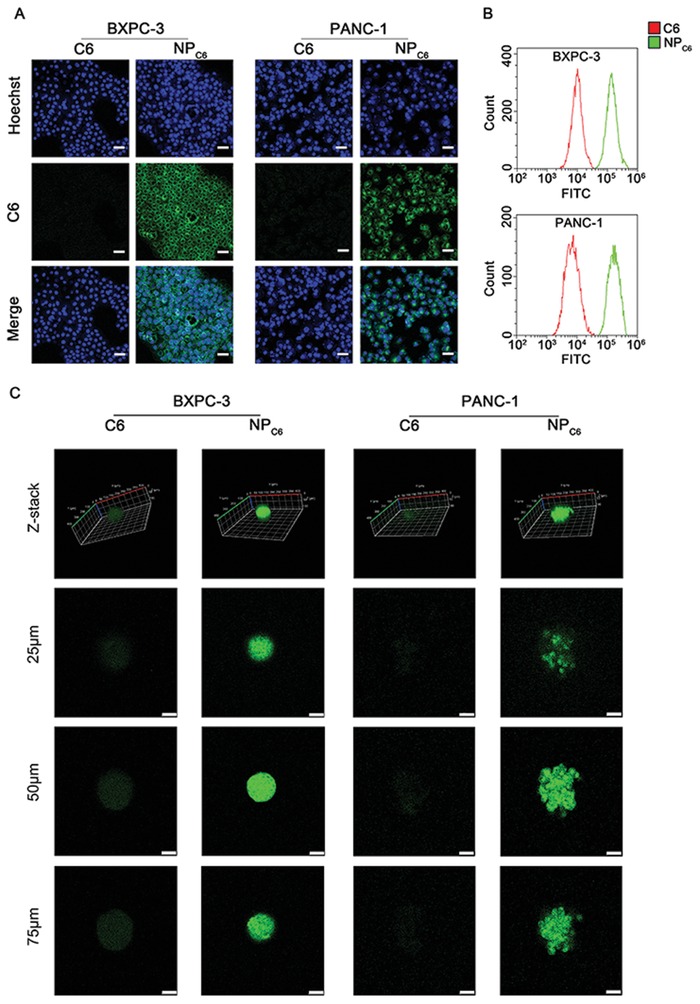
Cellular uptake behavior of J/T@8P4 NPs. A) Representative fluorescence images for the cellular uptake of C6 or NP_C6_ in BxPC‐3 and PANC‐1 cells after 24 h incubation. Scale bar = 10 µm. B) Flow cytometry analysis of the fluorescence intensity of C6 or NP_C6_‐treated BxPC‐3 and PANC‐1 cells at 24 h. C) Representative fluorescence images for the cellular uptake of C6 or NP_C6_ in BxPC‐3 and PANC‐1 tumor spheroids. Scale bar = 50 µm. All presented results are from three independent experiments.

The drug internalization and persistent retention of PDAC cells play an important role in the treatment. Therefore, 8P4 NPs loaded with C6 (NP_C6_) were used to investigate the cellular uptake of 8P4 NPs by PDAC cells. As shown in **Figure** **4**A,B, the NP_C6_ showed higher cellular uptake than its free drug counterpart at 2 h. This could be explained by the surface charge of these 8P4 NPs, as positively charged NPs usually interact with cell membranes stronger than free drugs. The data also demonstrated that NP_C6_ can be ingested by PDAC cells and internalized into the cytoplasm.

The uptake assay in the monolayer cells may not accurately determine the uptake behavior of the NPs in PDAC tumors.^[^[qv: 8]^]^ We next investigated the uptake of NP_C6_ and free C6 by BxPC‐3 and PANC‐1 tumor spheroids, which are more capable of simulating the tumor uptake of NPs in vivo. The effect on the spheroid uptake of NP_C6_ and free C6 was consistent with that on monolayer cells. The fluorescence intensity of NP_C6_ in BxPC‐3 and PANC‐1 tumor spheroids was markedly stronger than that in C6 group (Figure 4C), indicating that 8P4 NPs may help the drug penetrate into the tumor spheroids.

### 2.5 Biodistribution and Tumor Accumulation of the 8P4 NPs In Vivo

**Figure 5:**
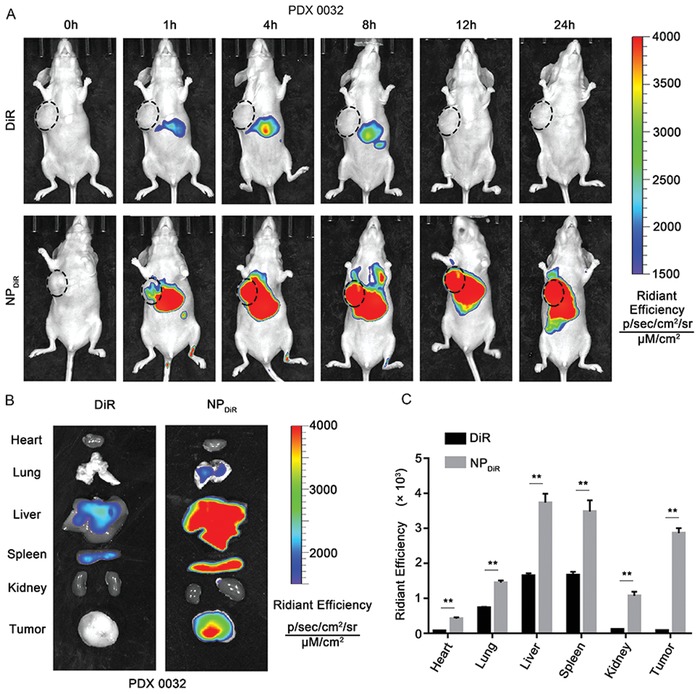
Biodistribution of the NPs in PDAC PDX model. A) Bioluminescence imaging of mice (*n* = 6) bearing PDX (PDX0032) following i.v. injection of free DiR or NP_DiR_. The images were taken at indicated time points after intraperitoneal injection of 2 mg of D‐Luciferin. B) Ex vivo fluorescence image of major organs and tumors from mice PDX (*n* = 6) treated with free DiR or NP_DiR_. C) Fluorescence quantitative assessment of luciferase expression in major organs and tumors. Data are presented as mean ± SD. *p < 0.05, **p < 0.01, and ***p < 0.001 were calculated according to a Student's *t*‐test. All presented results are from three independent experiments.

The biodistribution of 8P4 NPs was evaluated in the mice bearing subcutaneous PDX (PDX0032). The mice received intravenous (i.v.) injection of DiR loaded 8P4 NP (NP_DiR_) or free DiR. After the injection, each mouse was injected with D‐Luciferin at different time points intraperitoneally, and then imaged for bioluminescence (**Figure** **5**A). The fluorescence intensity of free DiR was accumulated in the liver, but little DiR was detected in the tumor. Notably, strong fluorescence intensity of NP_DiR_ was found in the tumor, indicating that the NPs exhibited significant tumor accumulation. 24 h post the injection, the main organs and tumors of mice in each group were obtained and imaged (Figure 5B). Consistently, we found that free DiR had no accumulation in the tumors. The NP_DiR_ generally led to higher tumor accumulation (Figure 5C). Fluorescence imaging of dissected tissues also showed that NP_DiR_ exhibited liver and spleen targeting. This can be explained by the fact that NPs tends to accumulate in the sinusoid‐rich tissues.

### 2.6 Enhanced Therapeutic Effects of J/T@8P4 NPs for Gemcitabine‐Resistant PDAC with Less Systemic Toxicity

**Figure 6:**
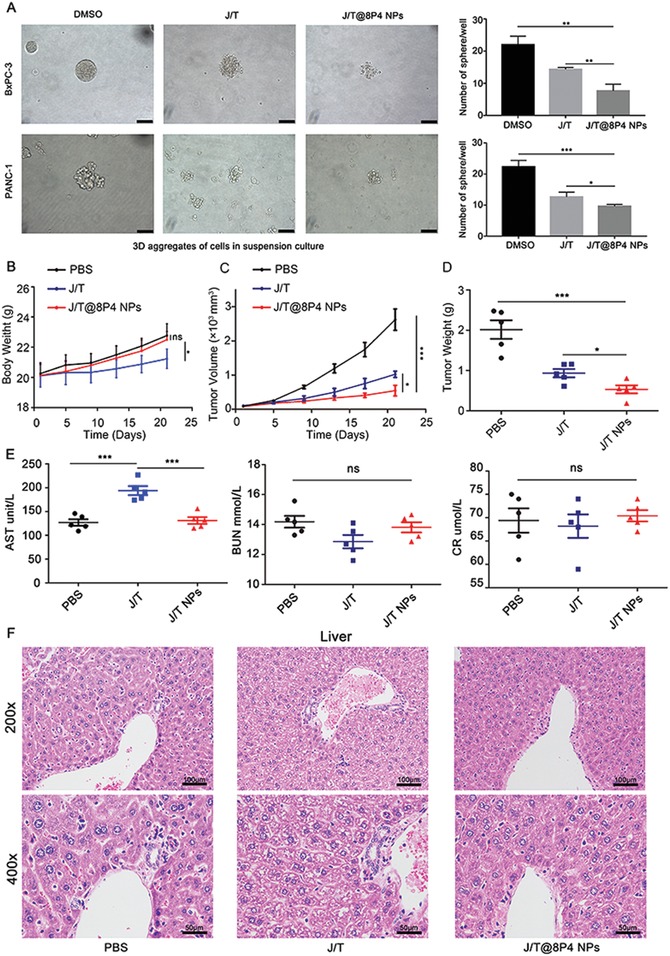
Tumor targeting efficiency of the 8P4 NPs in vivo. A) Tumor sphere formation assay of BxPC‐3 and PANC‐1 cells upon different treatment (DMSO, J/T, or J/T@8P4 NPs) on day 5. The sphere numbers were counted on day 7. B) PDX‐tumor (PDX0032) bearing mice were treated with PBS, J/T(JQ1 50 mg kg^−1^, daily; THZ1, 10 mg kg^−1^, twice daily), or J/T@8P4 NPs (JQ1 20 mg kg^−1^, THZ1, 10 mg kg^−1^, daily) for 21 days, respectively. Body weight of the mice (*n* = 5 each group) during the treatment period. C) Tumor growth curves of the mice (*n* = 5 each group). D) Weight of PDX tumors derived from the mice (*n* = 5 each group) in different group. E) Serum ALT, BUN, and CR from the mice (*n* = 5 each group). F) H&E staining of liver from the mice (*n* = 5 each group) in different group. Data are presented as mean ± SD. *p < 0.05, **p < 0.01, and ***p < 0.001 were calculated according to a Student's *t*‐test. All presented results are from three independent experiments.

Subsequently, we investigated the effects of different formulations on the tumor formation capacity of BxPC‐3 and PANC‐1 cells. We found that the number and size of colonies formed by J/T@8P4 NPs treated cells significantly reduced compared to control cells and combination of JQ1 and THZ1 treated cells (J/T) (**Figure** **6**A). Next, we proceeded to investigate whether J/T@8P4 NPs would exhibit anti‐PDAC efficacy with less systemic toxicity in a Gemcitabine‐resistant PDAC PDX model (PDX0032), which can mimic human PDAC growth for drug discovery.^[^[qv: 9]^]^ The PDX‐bearing mice were treated with J/T@8P4 NPs, J/T, or PBS for 21 days, respectively. No significant difference in body weight was observed between PBS group and J/T@8P4 NPs group (Figure 6B), whereas J/T treatment could significantly reduce the body weight of mice. As shown in Figure 6C,D, the tumor growth and weight of J/T group was significantly inhibited compared with PBS groups. Moreover, with the effect of 8P4 nanodelivery system on drug uptake and controlled release, the J/T@8P4 NPs treatment group showed the most significant antitumor efficacy. In addition, no apparent increase of AST, blood urea nitrogen (BUN), and creatinine (CR) in the serum was observed in J/T@8P4 NPs treatment group (Figure 6E). Histology analysis suggested severe edema of liver tissue in J/T group, but no histological alternations of liver (Figure 6F) and other organs (Figure S10, Supporting Information) were detected in J/T@8P4 NPs group compared with PBS group, indicating promising biocompatibility of J/T@8P4 NPs. Taken together, this SE‐associated oncogene targeting and 8P4‐based nanodrug will be an effective therapeutic strategy for PDAC treatment.

## 3 Discussion

Here, we demonstrate that the aberrant SE formation leads to upregulation of the active oncogene transcriptional program of PDAC cells, sensitizing PDAC to synergistic inhibition of BRD4 and CDK7, the two well‐known regulators of SE‐mediated transcription. This synergistic effect basically suppresses all active SE‐associated transcripts, especially those responsible for cell identity, resulting in cytotoxicity to normal cells, such as liver cells. Our proposed nanoenabled approach for initiating the SE‐targeting combinatorial therapy offers obvious advantages over current free drug strategies for PDAC. In this study, most of these evaluated nanodelivery approaches rely on the chemical properties of carrier materials and two drugs, and the limited loading efficiency of the NPs fails to lead a sufficient inhibition of PDAC cells. In contrast, the screened 8P4 NPs can effectively carry high amount of drugs with facilitated drug uptake and controlled drug release from lysosome, which may render the PDAC cells particularly susceptible to the induction of lysosomal‐mediated cell death. J/T@8P4 NPs could also be selective in tumors rather than migrate and accumulate in liver that are putatively required for improving targeting delivery and avoiding the major adverse effects of the combined therapy.

Although recent sequencing efforts have shown that PDAC tumors contain a high mutation rate, the mapping of mutations cannot guide the treatment of this fatal disease. Due to the misregulated TFs play dominant roles in driving and maintaining PDAC, we aimed to identify a candidate list of transcription factor genes associated with SEs. We assume that such a list will represent candidate oncogenic TFs in PDAC. Using ChIP‐seq against the H3K27ac in PDAC cells, we identified top‐ranked SE‐associated genes as proposed key candidate oncogenes in PDAC, which were highly vulnerable to THZ1 and JQ1 treatment. We further identified several SE‐associated oncogenic TFs in PDAC. One example is the transcription factor SMAD3, which represents a signaling effector of transforming growth factor‐β (TGF‐β) signaling.^[^[qv: 10]^]^ SMAD3 mediates transcription responsible for creating drug‐tolerant cancer stem‐like cells that facilitate metastatic progression.^[^[qv: 11]^]^ These findings suggest that transcriptional‐amplified SMAD3 may become a biomarker per se for THZ1/JQ1‐sensitive PDAC. EGFR, an important cell adhesion and signaling pathway mediator, is governed by SE in PDAC. EGFR is often overexpressed in many tumors, including lung cancer, breast cancer, colorectal cancer, and pancreatic tumors.^[^[qv: 12]^]^ Notably, EGFR inhibitor has been approved for the clinical treatment of lung and pancreatic cancer. EGFR‐mutated lung cancers are often sensitive to EGFR‐TKI.^[^[qv: 13]^]^ However, EGFR mutations are not common in PDAC. Erlotinib, an EGFR‐TKI, has shown only marginal benefit in PDAC without predictive biomarkers.^[^[qv: 14]^]^ Thus, identification of transcriptional‐amplified EGFR as a biomarker could benefit a subpopulation of patients who may be more likely to respond to Erlotinib treatment. Future work should investigate the role of HES1 and other SE‐associated TFs in the malignant transcriptional programs in PDAC.

The synergistic effects of the THZ1/JQ1 combination treatment on PDAC tumor were strongly correlated with incidence of cell cycle arrest and apoptosis. We believe that the synergism obtained due to simultaneous inhibition of BRD4 and CDK7 in SE complex might be an underlying mechanism for the enhanced antitumor effects. It should be noted that Gemcitabine, the standard treatment option for locally advanced and metastatic pancreatic cancer, showed merely modest antitumor effect compared with the THZ1/JQ1 combined therapy group. Previous investigations revealed that the failure of Gemcitabine clinical treatment can be attributed at least in part to development of Gemcitabine resistance in initially sensitive tumors within weeks of treatment. Our results here clearly showed that the THZ1/JQ1 combination effectively inhibited the growth of Gemcitabine‐resistant PDX, whose effects can be further enhanced by 8P4 nanodelivery system. It has been reported that Gemcitabine significantly upregulated expression of BRD4 in PDAC cell lines.^[^[qv: 15]^]^ In addition, BRD4 is crucial in PDAC tumorigenesis. These findings provide the rationale for using JQ1, the BRD4 inhibitor, as a therapeutic approach to treat the patients with Gemcitabine‐resistant PDAC.

“Smart” biological response materials sensitive to intracellular signals of cancer cells are attractive therapeutic platforms for the development of the next generation of personalized drugs.^[^[qv: 16]^]^ It has been reported that combretastatin A4 nanodrug boosted tumor‐selective release of Doxorubicin prodrug via inducing MMP9 amplification.^[^[qv: 17]^]^ Herein, we utilized the 8P4 as the most effective drug‐loading nanodelivery system to deliver THZ1/JQ1 into PDAC cells. Hydrophobic properties and unique chemical structure of 8P4 NPs contribute to the installation of high hydrophobic payloads. Indeed, in current study, accumulation of 8P4 NPs in lysosomes was also observed, and the polymer shells of 8P4 shedded in the lysosomes at 8 h post treatment. THZ1/JQ1 were then released into PDAC cells. Cancer cells have relatively large lysosomes, which are thought to be more vulnerable than regular‐sized lysosomes in normal cells. Additionally, cancer cells usually showed high metabolic rate and increased turnover of iron‐containing proteins, resulting in the lysosomal accumulation of iron. Consequently, cancer cells are sensitive to reactive oxygen species‐induced lysosomal membrane permeabilization (LMP).^[^[qv: 18]^]^ Therefore, based on these facts, we propose that during internalization the J/T@8P4 NPs accumulated in the fragile lysosome of PDAC, and then the breakdown of 8P4 NPs impaired the lysosomes leading to the LMP and release of THZ1/JQ1 for enhanced therapeutic efficacy in PDAC cells.

## 4 Conclusion

In conclusion, by comprehensively charting the epigenetic landscape of SE in PDAC cell lines, we identified a list of SE‐associated TFs, which drive the oncogenic transcriptional programs that specify PDAC cell characteristics. Moreover, we use the screened 8P4 NPs to deliver a synergistic combination of SE inhibitors further demonstrating the potency of targeting the oncogenic transcriptional amplification for PDAC treatment. Such a THZ1/JQ1‐loaded nanodrug can be a powerful agent to inhibit multiple oncogenic pathways simultaneously with less side‐effects compared with free SE inhibitors. The data presented here demonstrate that the THZ1/JQ1 nanodrug is a promising therapeutic strategy for Gemcitabine‐resistant PDAC treatment. And utilizing nanocarrier to deliver drugs targeting SEs opened a new door for cancer therapy.

## 5 Experimental Section

##### PDAC Cell Lines

The PDAC cell lines (BxPC‐3, PANC‐1, and SW‐1990) were purchased from the Cell Resources Center of Shanghai Institutes for Biological Science, Chinese Academy of Sciences (Shanghai, China). BxPC‐3 cells were cultured in RPMI 1640 (Gibco, USA) supplemented with 10% of FBS; PANC‐1 cells were cultured in high glucose Dulbecco's modified Eagle's medium (Gibco, USA) with 10% of FBS; and SW‐1990 cells were cultured in Leibovitz's L‐15 medium (Gibco, USA) supplemented with 10% of FBS. All cell lines were cultured at 37 °C and 5% CO_2_ in a humidified atmosphere.

##### Antibodies and Chemicals

The H3K27ac antibody (Catalog number: ab4729) was purchased from Abcam. Anti‐rabbit IgG (Catalog number: 7074) was purchased from Cell Signaling Technology. JQ‐1 (Catalog number: S7110) and THZ1 (Catalog number: S7549) were purchased from Selleckchem (Houston, TX, USA). Gemcitabin (Catalog number: T6069) was purchased from TopScience (Shanghai, China). Toluene‐4‐sulfonic acid monohydrate, 1,4‐butanediol, sebacoyl dichloride, L‐phenylalanine, sebacoyl dichloride, toluene, p‐nitrophenol, triethylamine, acetone, and ethyl acetate were purchased from Aladdin.

##### Chromatin Immunoprecipitation (ChIP) Sequencing (ChIP‐seq) and Analysis

ChIP with antibodies H3K27ac (Abcam, ab4729) were conducted with the Magna ChIP Kit (Millipore, USA) according to manufacturer instructions. ChIP‐seq libraries were sequenced on the HiSeq 2000 platform supplied by Novogene (Beijing, China). SEs were identified using the rank ordering of super enhancers (ROSE) algorithm. SEs had two or more H3K27ac peaks (detected by MACS2) within a range of 12 kb, and exceeded 2.5 kb from the transcriptional start site. SEs were further defined as those with the highest level of acetylation of H3K27 by graphing an inflection plot and selecting values for which the slope of a fitted curve exceeded the value of 1. The enhancers below the point on that curve with a slope of 1 were classified as TEs.

##### RNA‐Sequencing (RNA‐seq) and Analysis

The total RNA was isolated with the TRIzol reagent (Invitrogen). The construction of cDNA library was completed by Novogene (Beijing, China). The sequencing was performed using Illumina HiSeq 2500 platform supplied by Novogene. An R package, DESeq, was applied for transcription quantification and differential expression analysis using a cutoff of *p* < 0.05. GO analysis was performed by clusterProfiler package in R software. Pathway analysis was conducted utilizing the GSEA supported by the Broad Institute website (https://www.broadinstitute.org/gsea/index.jsp).

##### Cell Viability and Drug Synergistic Effect Calculations

For cell viability assay, cells were seeded into 96‐well plates in at least triplicates. Cell viability was measured at 24, 48, and 72 h after treatments with the CellTiter‐Glo Luminescent Cell Viability Assay (CTG assay) kit (Promega) according to the manufacturer instructions. For testing synergistic effect of THZ1 and JQ1, the cells were treated with each drug alone or in combination, and then the cell viability was determined by CTG assay. CI was calculated using CalcuSyn software (Biosoft). The CI less than 1.0 was considered to be synergistic effect.

##### Cell Cycle and Apoptosis Assays

Cell cycle analysis was performed using the Cell Cycle staining kit (CCS012, Multi Sciences) according to manufacturer instructions. Cell apoptosis was measured with Annexin V‐FITC/PI Apoptosis Detection Kit according to the manufacturer protocol (AP101‐100‐kit, Multi Sciences). The stained cells were collected for analysis on CytoFLEX (Beckman Coulter), and the data were analyzed with CytExpert software.

##### Invasion and Migration Assays

Cell invasion and migration arrays with or without Matrigel (Corning, NY, USA) were used to assess tumor cell invasion and migration abilities. ≈4 × 10^4^ cells in 300 µL medium without FBS were seeded in transwell chambers with (invasion assay) or without (migration assay) Matrigel. The lower chamber was added with 800 µL medium with 10% FBS. After 24 h of culture, the cells attached to the lower surface of the membrane were fixed with 4% formaldehyde and stained with 0.5% crystal violet. Five random fields of the membrane were counted under a microscope.

##### Formation of 3D Tumor Spheroids

To prepare 3D tumor spheroids, PANC‐1 and BxPC‐3 cells were seeded in the Corning 96 Well Ultra Low Attachment Microplate at a density of 2 × 10^3^ cells per well. After 5 days, the compacted tumor spheroids were treated with different drug formulations. On day 7, the morphology and number of the spheroids were recorded under an inverted microscope.

##### Subcutaneous Xenograft Model of PDAC

BxPC‐3 and PANC‐1 cells (10^7^) in 150 µL PBS were subcutaneously injected into the right flank of nude mice. Tumor volume was measured by means of a caliper and calculated as length × width^2^/2. When tumor volume grew up to 50–100 mm^3^, the mice were randomly divided into five groups, and then treated with PBS daily, Gemcitabine (50 mg kg^−1^, twice per week), JQ1 (50 mg kg^−1^, daily), THZ1 (10 mg kg^−1^, twice daily), or JQ1 (50 mg kg^−1^, daily) combined with THZ1 (10 mg kg^−1^, twice daily). Tumor volume was measured every 4 days. 21 days after implantation, the mice were euthanized according to the protocol filed by the Guidance of Institutional Animal Care and Use Committee (IACUC) of Sun Yat‐Sen University. The concentration of the serum AST, CR, and BUN was measured. The tumor xenografts were excised, fixed, weighed, photographed, and stored. All the animal experiments were carried out with the approval of the Institutional Review Board of the First Affiliated Hospital of Sun Yat‐Sen University ([2019] No. 124).

##### PDX Model

Mice bearing passage 3 PDAC PDX (PDX0032) were randomly divided into three groups, and then treated with PBS daily, JQ1 (50 mg kg^−1^, daily) combined with THZ1 (10 mg kg^−1^, twice daily) or JQ1/THZ1@8p4 NPs daily (JQ1 20 mg kg^−1^, THZ1 10 mg kg^−1^). Tumor volume was measured every 4 days. 17 days after implantation, the mice were euthanized according to the protocol filed by the Guidance of Institutional Animal Care and Use Committee (IACUC) of Sun Yat‐Sen University. The concentration of the serum AST, CR, and BUN was measured. The tumor xenografts were excised, fixed, weighed, photographed, and stored. Mice organs (heart, lung, liver, spleen, and kidney) from each group were paraffin‐embedded for hematoxylin and eosin (H&E) staining. All the animal experiments were carried out with the approval of the Institutional Review Board of the First Affiliated Hospital of Sun Yat‐Sen University ([2019] No. 124).

##### Preparation and Characterization of JQ1/THZ1@8p4 NPs

The synthesis of 8‐Phe‐4(8P4) polymers was described in the previous paper.^[^[qv: 19]^]^ 8p4 and DSPE‐PEG_2000_ were dissolved in dimethyl sulfoxide (10 mg mL^−1^), then added dropwise the mixture to stirring water to prepare NPs (Figure S2, Supporting Information). Dynamic light scattering (DLS, Malvern Zetasizer Nano‐ZS90) was used to measure the particle size. To observe the morphology of NPs, 5 µL of each sample was placed on the grid and negatively stained by uranium acetate, and then observed by transition electron microscopy (TEM, JEM‐1400 Plus, 120 kV, JEOL). For stability testing, different NPs were exposed to PBS (pH 7.4) or PBS (pH 7.4) containing 10% v/v FBS. Then, the particle size was measured at different time points. The drug loading capacity and encapsulation efficiency were measured by high‐performance liquid chromatography (HPLC; Agilent 1260 Infinity II) as following formulas

(1) $$\text{LC}\;(\%)\;\text{=}\;\frac{\text{the}\;\text{weight}\;\text{of}\;\text{encapsulated}\;\text{drug}}{\text{the}\;\text{total}\;\text{weight}\;\text{of}\;\text{nanoparticles}}$$

(2) $$\text{EE}\;(\%)\;\text{=}\;\frac{\;\text{the}\;\text{weight}\;\text{of}\;\text{encapsulated}\;\text{drug}}{\text{the}\;\text{total}\;\text{weight}\;\text{of}\;\text{drug}}$$

##### In Vitro Drug Release Test

Dialysis method was used to determine the drug release behavior of JQ1/THZ1@8p4 NPs. Briefly, JQ1/THZ1@8p4 NPs solution was transferred in a 3500 kDa dialysis bag, and then exposed to different pH conditions (pH = 5.0 and pH = 7.4) at 37 °C. Then, 500 µL of the release medium was withdrawn and replaced with fresh medium at different time points. The amount of drug release from JQ1/THZ1@8p4 NPs was measured by HPLC.

##### Cellular Uptake Assay

BxPC‐3 and PANC‐1 cells (1.5 × 10^5^ cells per well) were seeded in a six‐well plate or in a confocal dish for 24 h. In order to determine drug uptake of spheroids, BxPC‐3 and PANC‐1 cells were seeded in a Corning 24 Well Ultra Low Attachment Microplate at a density of 2 × 10^3^ cells per well for 5 days. The cells or spheroids were incubated with the Coumarin 6 (2 µg mL^−1^) loaded 8P4 NPs for 2 h. The cellular uptake efficiency was determined by confocal laser scanning microscopy (CLSM).

##### Intracellular Distribution of NPs in PDAC cells

The BxPC‐3 cells (1.5 × 10^5^ cells per well) were seeded in a confocal dish and cultured for 24 h. After washing with PBS, the cells were incubated with Coumarin 6 loaded 8P4 NPs (2 µg mL^−1^) at 37 °C for 1, 4, or 8 h. Next, the cells were incubated with 1 mL Hoechst 33342 (5 µg mL^−1^) at 37 °C for 10 min, and then incubated with Lyso‐Tracker Red (1 mL, 100 × 10^9^ m) for another 10 min. Subsequently, the cells were fixed with 4% formaldehyde for CLSM (Olympus, Japan) observation.

##### Biodistribution of NPs by In Vivo Imaging System (IVIS)

Mice bearing passage 3 PDAC PDX (PDX0032) were randomly divided into two groups with three in each group. After intravenous injection of the free DiR (0.5 mg kg^−1^) and DiR‐loaded NPs (the equivalent DiR dose is 0.5 mg kg^−1^), the whole‐body fluorescence distribution patterns were obtained at 1, 4, 8, 12, and 24 h using the IVIS imaging system (PerkinElmer, USA). After euthanasia of the mice, the tumors and main organs (heart, lung, liver, spleen, and kidney) were collected for ex vivo imaging. The fluorescence intensity of the isolated organs and tumor tissues was quantitatively evaluated.

##### Statistical Analyses

All the experiments were repeated at least three times. Unpaired Student's *t*‐test, chi square test, or one‐way analysis of variance were used to determine the significance of differences between groups. Continuous variables were expressed as mean ± SD. In all the statistical analyses, *p* < 0.05 was considered as statistically significant.

## Conflict of Interest

The authors declare no conflict of interest.

## Supporting information

Supporting Information
