# Supplementary material for: Targeting Super‐Enhancers via Nanoparticle‐Facilitated BRD4 and CDK7 Inhibitors Synergistically Suppresses Pancreatic Ductal Adenocarcinoma
Source: Adv Sci (Weinh). 2020 Feb 16;7(7):1902926. doi: 10.1002/advs.201902926 (PMC7140991; doi:10.1002/advs.201902926)
Supplement: Supplementary file 1 — Supporting Information [file ADVS-7-1902926-s001.pdf]

Copyright WILEY-VCH Verlag GmbH & Co. KGaA, 69469 Weinheim, Germany, 2020.

ADVANCED SCIENCE
Open Access

Supporting Information

for *Adv. Sci.*, DOI: 10.1002/advs.201902926

Targeting Super-Enhancers via Nanoparticle-Facilitated BRD4 and CDK7 Inhibitors Synergistically Suppresses Pancreatic Ductal Adenocarcinoma

Chen-Song Huang, Xinru You, Chunlei Dai, Qiong-Cong Xu, Fuxi Li, Li Wang, Xi-Tai Huang, Jie-Qin Wang, Shi-Jin Li, Zhuoxing Gao, Jun Wu, Xiao-Yu Yin,* and Wei Zhao**

Supplementary Information

Targeting Super-Enhancers via Nanoparticle-facilitated BRD4 and CDK7 Inhibitors Synergistically Suppresses Pancreatic Ductal Adenocarcinoma

Chen-Song Huang[#], Xinru You[#], Chunlei Dai[#], Qiong-Cong Xu[#], Fuxi Li, Li Wang, Xi-Tai Huang, Jie-Qin Wang, Shi-jin Li, Zhuoxing Gao, Jun Wu, Xiao-Yu Yin*, and Wei Zhao**

Contents

Figure. S1-S10

Table S1-S2

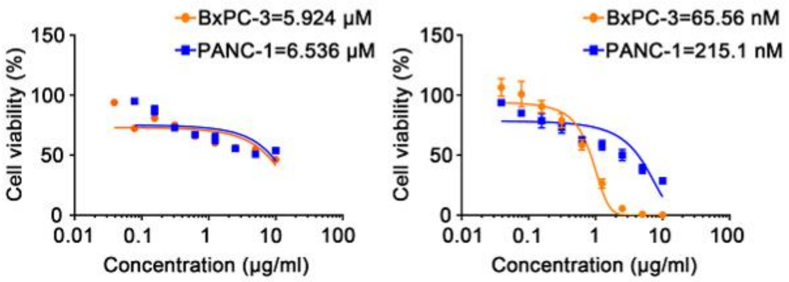

Figure S1. Synergistic effects of JQ1 and THZ1 against PDAC

BxPC-3 and PANC-1 cells were treated with JQ1 (left) or THZ1 (right) for 48 h at indicated concentrations. The cell viability relative to DMSO-treated cells was shown.

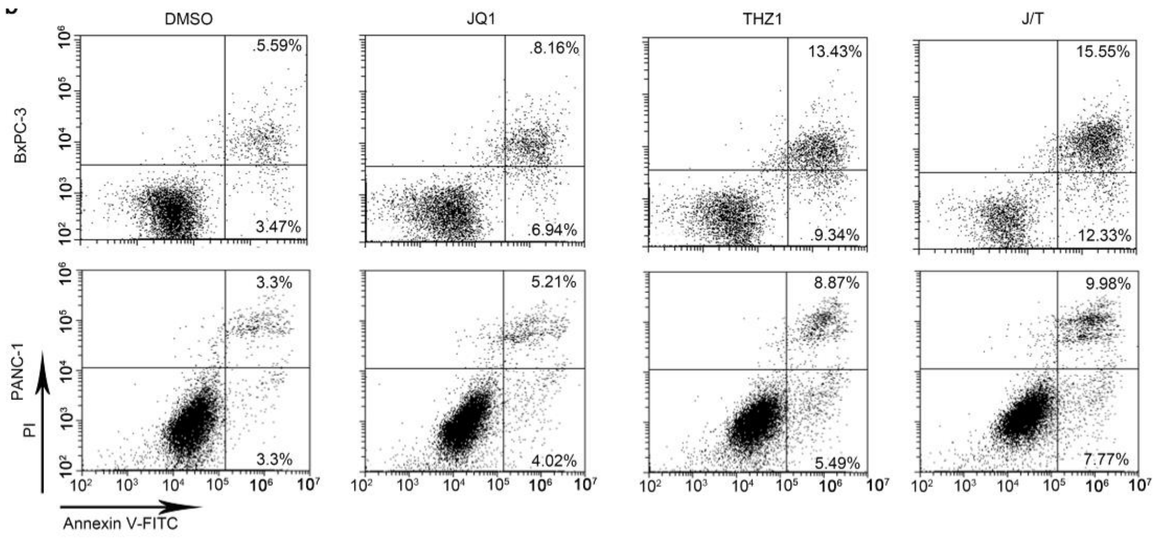

Figure S2. Apoptosis analysis in BxPC-3 and PANC-1 cells treated with JQ1 and THZ1 individually or in combination.

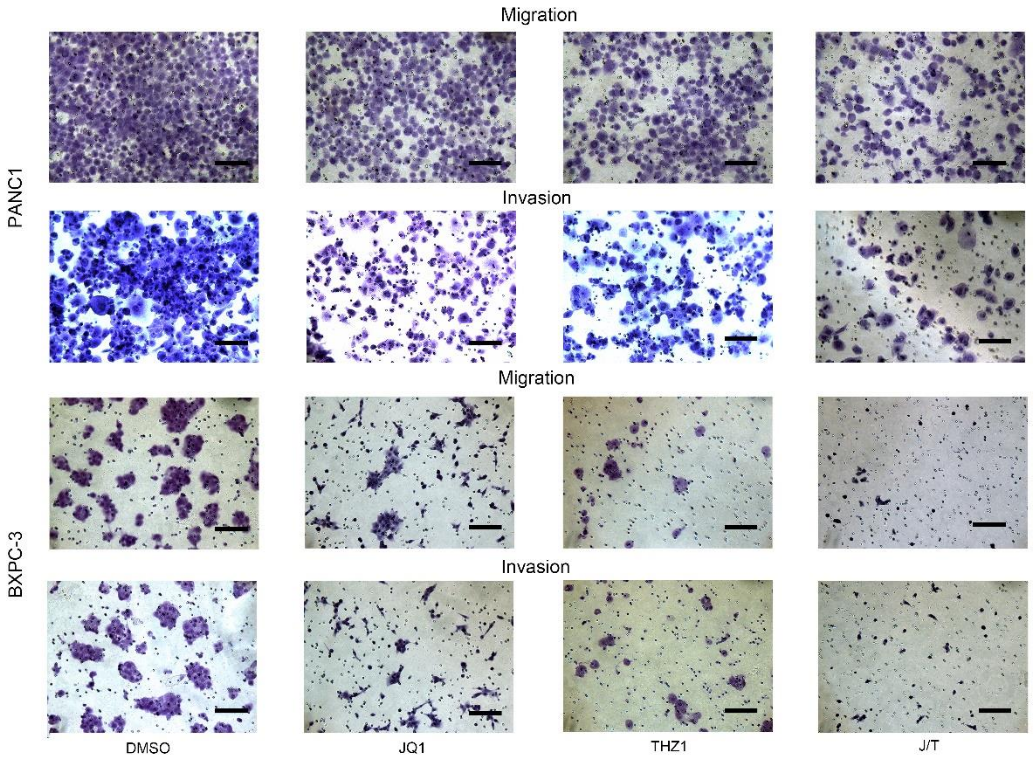

Figure S3. Invasion and migration assays of BxPC-3 and PANC-1 cells after treatment with JQ1 and THZ1 individually or in combination. The cells which reached the bottom of membranes were photographed (200× magnification).

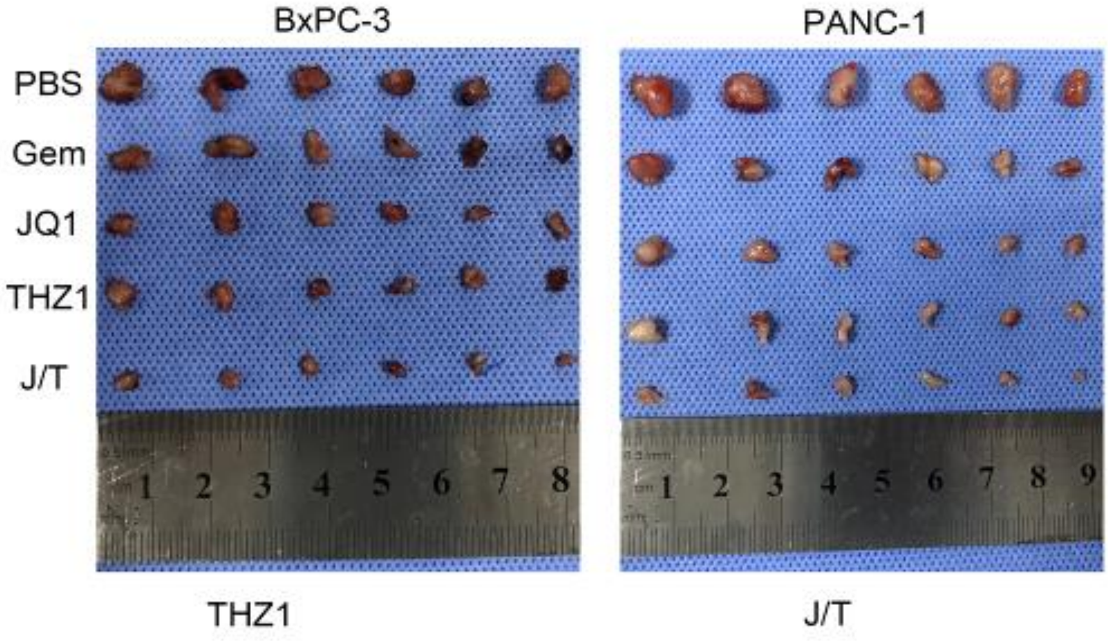

Figure S4. Images of tumors derived from the mice (n=6) in different groups. The mice (n=6 per group) were treated with PBS, Gemcitabine (Gem, 50 mg/kg, twice per week), JQ1 (50 mg/kg, daily), THZ1 (10 mg/kg, twice daily), and JQ1 (50 mg/kg, daily) combined with THZ1 (10 mg/kg, twice daily) for 21 days.

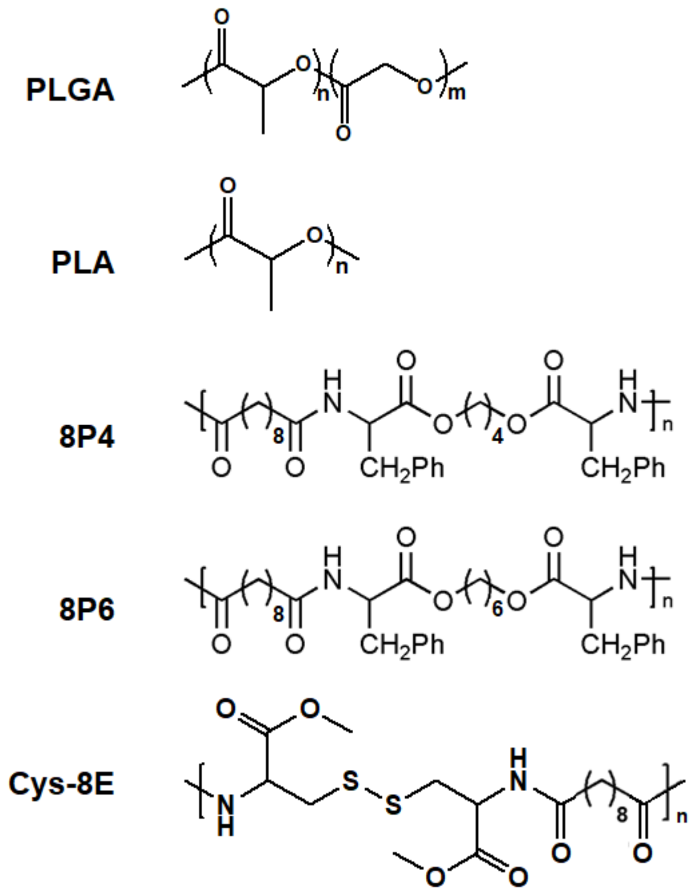

Figure S5. The structure of different polymers.

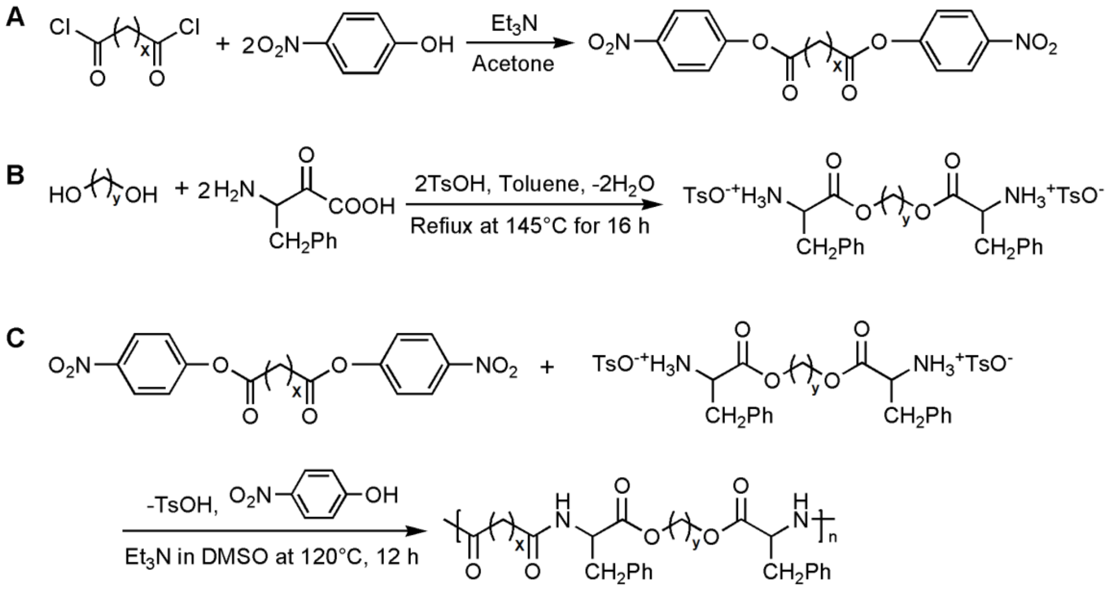

Figure S6. The synthetic routes of x-Phe-y polymers. (8p4 means x=8, y=4).

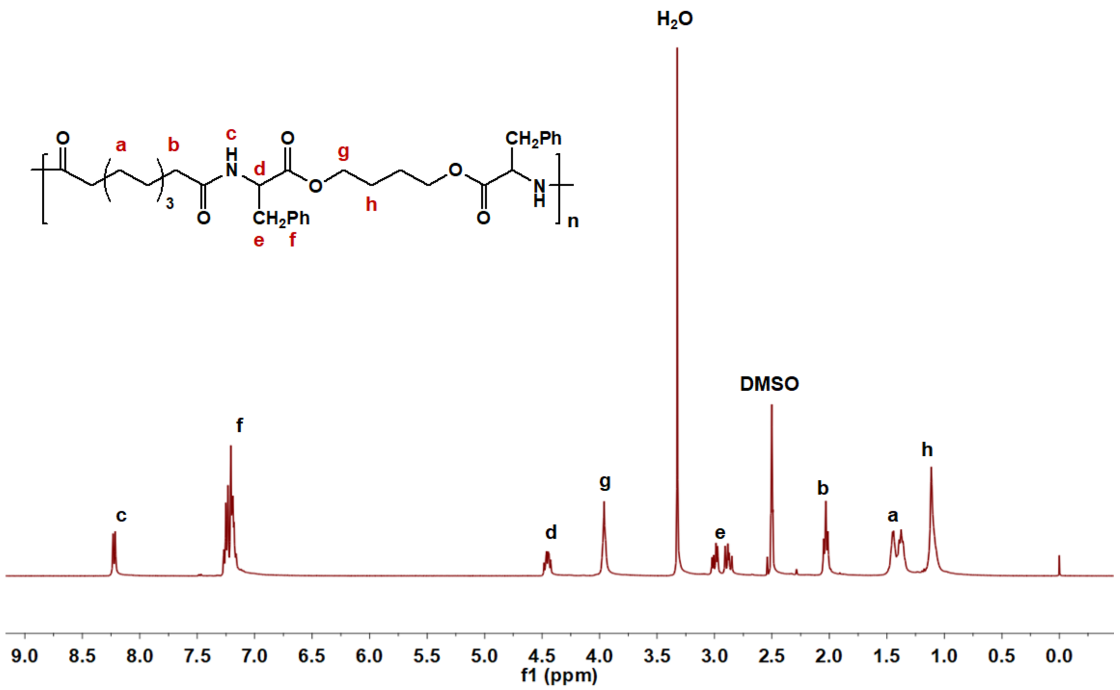

Figure S7. ^{1}H-NMR (Nuclear magnetic resonance) spectrum of 8p4 polymer.

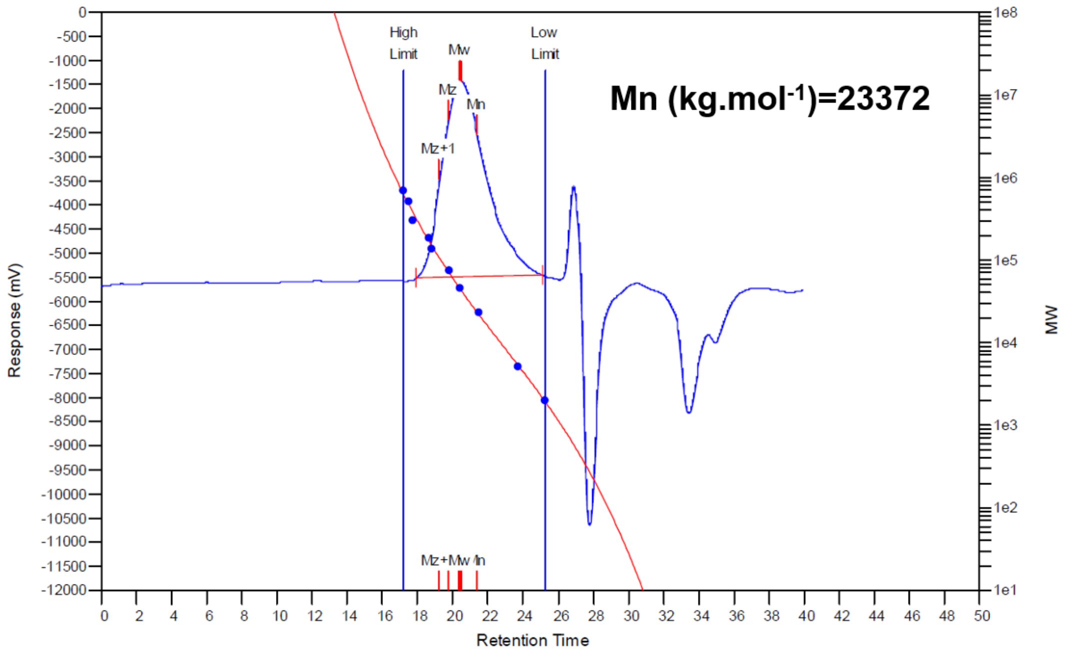

Figure S8. Gel permeation chromatography (GPC) spectrum of 8p4 polymer.

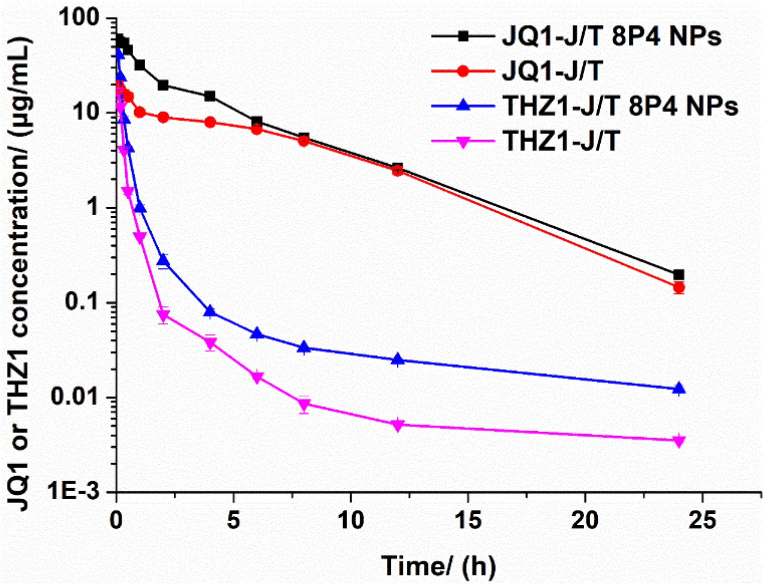

Figure S9. Pharmacokinetics of J/T@8P4 NPs.

SD rats were randomly divided into two groups (n=3) and intravenously injected with J/T@8P4 NPs or JQ1/THZ1 at a 20 mg/kg JQ1 equivalent dose and 10 mg/kg THZ1 equivalent dose. Blood samples were collected from the retroorbital plexus at a indicated time points. Heparinized plasma was collected by centrifugation at 3,500 rpm for 15 min at 4 °C. The supernatant was stored at -80 °C until analysis. The concentrations of JQ1 and THZ1 were determined by high performance liquid chromatography-mass spectrometry (HPLC-MS).

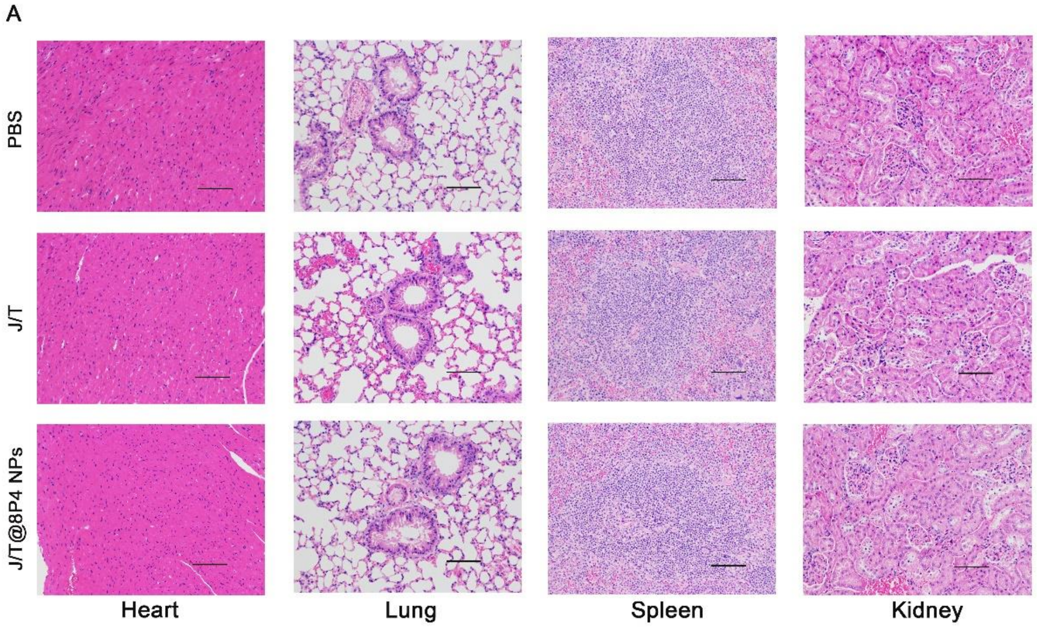

Figure S10. Hematoxylin and eosin (H&E) staining of main organs (Heart, Lung, Spleen and Kidney) of indicated groups was shown. (Scale bar=150 μm)

Table S1. Maximum drug-loading range of polymers by nanoprecipitation

Polymer	*8P4*	*8P6*	*Cys-8E*	*PLGA(75:25)*	*PLGA(50:50)*	*PLA*
JQ1 loading (%)	**≤18**	**≤10**	**≤20**	**≤8**	**≤10**	**≤8**
THZ1 loading (%)	**≤8**	**≤4**	**≤3**	**≤2**	**≤2**	**≤2**

Table S2. Optimization of polymer-drug ratio for efficient drug loading in different NPs.

	Polymer /drug (w/w)	Size (nm)	PDI	JQ1		THZ1	
				LC%	EE%	LC%	EE%
**J/T@8P4 NPs**	9/1	124.50±0.67	0.18	2.18	26.41	1.30	74.05
	5/1	111.0±0.46	0.112	4.74	34.45	2.35	85.50
	2/1	92.85±1.03	0.133	7.96	28.65	3.97	71.50
	1/1	89.50±2.53	0.19	16.57	66.29	7.44	89.15
**J/T@Cys-8E NPs**	9/1	141.85±0.64	0.125	4.08	49.45	0.62	35.35
	5/1	123.15±6.43	0.147	1.28	9.33	0.31	11.30
	2/1	135.6±2.26	0.16	11.89	42.85	1.71	31.06
	1/1	172.95±6.29	0.17	19.53	46.77	2.76	33.51
